# Kilner’s Druggist Formulary

**Published:** 1881-10-20

**Authors:** 


*Kilner's Druggist Formulary.*—We have the last quarterly supplement of this work. We regard it as the best thing of the kind now extant. See the advertisement in this Journal.

